# A Comprehensive Review of the Effect of Honey on Human Health

**DOI:** 10.3390/nu15133056

**Published:** 2023-07-06

**Authors:** Marta Palma-Morales, Jesús R. Huertas, Celia Rodríguez-Pérez

**Affiliations:** 1 Biomedical Research Centre, Institute of Nutrition and Food Technology (INYTA) ‘José Mataix’, University of Granada, Avda. del Conocimiento s/n, 18071 Granada, Spain; martapalm@ugr.es (M.P.-M.); jhuertas@ugr.es (J.R.H.); 2 Department of Nutrition and Food Science, Faculty of Pharmacy, University of Granada, Cartuja Campus, 18011 Granada, Spain; 3 Primary Care Promotion of Maternal, Child and Women’s Health for Prevention of Adult Chronic Diseases Network (RD21/0012/0008), Institute of Health Carlos III, 28029 Madrid, Spain; 4 Instituto de Investigación Biosanitaria ibs.GRANADA, 18012 Granada, Spain

**Keywords:** honey, health, clinical trials, cough, cancer, hyperlipidemia, diabetes, gastroenteritis

## Abstract

Honey is a nutritious, healthy, and natural food, to which antioxidant, anti-inflammatory, and antimicrobial properties have been attributed, mainly due to its content of phenolic compounds. The aim of this review is to analyze the available evidence of the effect of honey on humans. Forty-eight clinical trials published between 1985 and 2022 were analyzed, with a total of 3655 subjects. More beneficial effects of honey intake than no or negative effects on different cardiovascular and metabolic risk factors, glucose tolerance, mucositis caused by chemo-radiotherapy, cough in children and wound healing, among others have been observed. Although the number of studies conducted to date is limited and the different investigations are not standardized, beneficial effects of honey intake have been observed, especially when its intake replaces the intake of other sweeteners. In addition, honey could be a safe adjuvant to be administered alongside drugs used for certain diseases.

## 1. Introduction

Honey is a natural substance produced by honey bees (*Apis mellifera*). They collect flower nectar, plant secretions or excretions of plant-sucking insects from plants and transform it into honey [1]. Worldwide, 1779.6 metric tons of honey are produced, and the market value of honey is expected to grow by 2028 [2]. China produces almost 28% of the world’s honey, followed by Turkey (5.9%), Iran (4.5%), the United States (4.1%), and India (3.5%) [3]. The main exporters of honey are China, New Zealand, Argentina, Germany, Ukraine, India, and Spain while the United States, Germany, Japan, France, the United Kingdom, Italy, and China lead the import [3].

**Figure 1:**
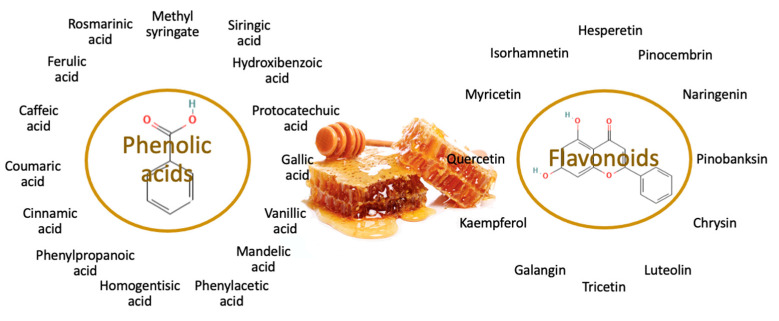
Phenolic compounds characterized in honey.

Honey is considered a nutritious, healthy, and natural food, whose composition is highly variable depending on its botanical and geographical origin [4]. It is mainly composed of a mixture of different sugars (80–85%), water 15–17%, and proteins (0.1–0.4%) [5], but it also contains enzymes, organic acids, vitamins, minerals, and phenolic compounds to a lesser extent, which contribute greatly to its sensory and functional characteristics [5]. The color can range from white to brown and is largely determined by the presence of phenolic compounds and minerals [5]. Honey is classified according to its botanical origin as monofloral—when it is produced from the nectar or honeydew of a single botanical species or if its presence is predominant and multifloral—when it comes from more than one botanical species [6]. Antioxidant, anti-inflammatory, antibacterial, and antiviral properties have been linked to honey intake which has contributed to increasing the interest in this food [4]. Despite the fact that bioactive compounds implicated in those effects have not been fully elucidated, the beneficial effects of honey on human health have been attributed to its content of phenolic compounds [7]. Phenolic compounds are synthesized by plants under normal and stress conditions [8] and have several functions, such as attracting insects for pollination and protecting against pathogens and ultraviolet radiation, among others [9]. They are characterized for having one or more aromatic rings with one or more hydroxyl groups. Its content varies depending on the variety, origin, agronomic and storage conditions, harvest time, and climate [10]. In fact, recent research reported that values of total phenolic content (TPC) from different honeys ranged between 0.65 ± 0.42 and 84.17 ± 30.40 mg/100 g [11]. Among them, the majority of the characteristics are flavonoids and phenolic acids. In this regard, Figure 1 shows the main phenolic compounds characterized in honey. Phenolic compounds, in addition to being considered as bioactive compounds, can act as biomarkers of honey origin or adulteration [1].

Considering the interesting properties that have traditionally been attributed to honey intake and the fact that most of the studies supporting those properties have been carried out in vitro or in animal models, the aim of this review is to analyze the available evidence of the effect of honey on humans. In this paper, the effects of honey on human health are presented from a comprehensive approach, including the clinical trials published from 1985 to the present.

## 2. Materials and Methods

The US National Library of Medicine National Institutes of health (PubMed) and Web of Science databases have been employed for conducting literature searches from 1985 to 2022 to achieve an overview of all the available evidence regarding the effects of honey on human health. Different combinations of the following keywords were used: Honey, health, diabetes, metabolic cardiovascular syndrome, obesity, cancer, upper respiratory infections, and antimicrobial. In PubMed, we used the following search equation strategy: “honey” [All Fields] AND (“health” [All Fields] OR “diabetes” [All Fields] OR “metabolic cardiovascular syndrome” [All Fields] OR “obesity” [All Fields] OR “cancer” [All Fields] OR “upper respiratory infections” [All Fields] OR “antimicrobial” [All Fields]). When we used PubMed, we included Medical Subject heading (MeSH) terms to increase the power of the search. In addition, 4613 results were obtained, then filtered by “full text” (4348 results), “clinical trial” (131 results), and “English language” (130 results). The search equation used in Web of Science was: Honey AND (health OR diabetes OR metabolic cardiovascular syndrome OR obesity OR cancer OR upper respiratory infections OR antimicrobial). Moreover, 734 results were obtained, then filtered by “open access” (375 results), “article” (301 results), and “English language” (299 results).

The main criteria of PICO (Population, Intervention, Comparison, Outcome) were followed to frame and answer the clinical-related question. In this regard, the population included humans (healthy subjects, subjects who are overweight or obese, diabetes, cancer, wounds, infections, and inflammation), the intervention was treatment with different types of honey, oral or topical, without combining it with other substances, and the comparison was made between honey and other sugar sweeteners. The outcome was cardiovascular, anticancer, antidiabetic, antimicrobial and antiviral, anti-obesity and antioxidant effects.

The inclusion criteria were the following: (1) Clinical trials with honey; (2) food and nutrition-related studies; (3) studies written in English. The exclusion criteria were: (1) Review articles; (2) studies written in languages other than English; (3) studies without controls; (4) studies without full access. Once the articles were selected based on reading the title, abstract, and full text, duplicates were removed and the quality of the clinical trials was assessed based on the PEDro scale (https://pedro.org.au/spanish/resources/pedro-scale/ (accessed on 21 June 2023)), which evaluates the infernal validity as well as the statistical information to establish that the results are interpretable. Then, the results were classified according to the different health effects attributed to honey, i.e., effect on healthy subjects, subjects who are overweight or obese, diabetes, cancer, abscesses, wounds, blepharitis, rhinoconjuntivitis, children with upper respiratory tract infections (URTIs) or gastroenteritis, and women with vulvovaginal candidiasis and dysmenorrhoea. Those data have been summarized in different tables along the text and a narrative review of the main outcomes has been included.

## 3. Results

Forty-eight articles published in 42 different journals were analyzed, with a total of 3655 subjects with 29.51 ± 21.51 years of age, of whom 1990 consumed or were treated with honey. Of the 3655 subjects, at least 1803 were women (two studies did not specify). The studies included different population groups (healthy subjects, overweight or obese subjects, diabetic subjects, subjects with cancer, children, etc.) and included more than 30 different types of honey. Although it is not a systematic review, the results of the PEDro scale regarding the quality of the articles were in the range of 6–10, with articles scoring 6 or higher being considered of good methodological quality.

### 3.1. Cardiovascular and Metabolic Risk Factors

Table 1 shows the studies found on the effects of honey on different cardiovascular or metabolic risk factors in different population groups, i.e., healthy, overweight or obese, diabetic and hyperlipidemic subjects. 

#### 3.1.1. Healthy Subjects

Several authors have associated honey consumption with improvements of lipid profile in healthy subjects. In this regard, a supplementation with 70 g/day of honey for 6 weeks significantly improved the lipid profile of young men (18–30 years) compared to subjects supplemented with sucrose [12]. Specifically, triglycerides (TG), total cholesterol (TC), and low-density lipoproteins (LDL) levels decreased, and high-density lipoprotein (HDL) levels increased between the beginning and end of the study. Those differences were also significant compared to the control group. A significant improvement in lipid profile were also demonstrated in a study conducted on young Pakistani men (20.13 ± 0.14 y) [13]. In the experimental group supplemented with 70 g/day of natural, unprocessed honey purchased from Ilyas Traders, Charsadda, Khyber Pakhtunkhwa, (Pakistan) for 4 weeks, a significant decrease in total and LDL cholesterol levels was observed, while the HDL level increased significantly. However, changes in fasting blood glucose (FBG) and TG levels were not significant. When the two groups were compared, the increase in FBG in the experimental group was significantly lower than in the control group, the decrease in TG, TC, and LDL levels and the increase in HDL level in the experimental group were also significant compared to the control group. In addition, oral glucose tolerance (OGT) was significantly higher after honey consumption than after glucose consumption. Contrarily, Al-Tamimi et al. [14] showed no significant effects on lipid profile or basal insulin when supplemented with 1.5 g/kg/day of a mixture of four types of clover honey obtained from Golden Heritage Foods, Smitty Bee Honey, Millers Honey Company, and Marshall’s Farm Natural Honey, for 1 month in healthy subjects aged 24–57 years. Interestingly, honey consumption did not produce the negative responses in TG levels that sucrose intake did, and thus suggests that the substitution of sucrose with natural honey may be beneficial. These results seem to indicate that supplementation for 6 weeks has a greater effect than for 4 weeks.

#### 3.1.2. Overweight or Obese Subjects

Yaghoobi et al. [15] conducted a study on overweight/obese subjects in which the experimental group consumed 70 g/day of Iranian natural honey and the control group consumed the same amount of sucrose for a month. Honey consumption resulted in a significant reduction in body mass index (BMI) and FBG. Moreover, honey significantly reduced serum TG and C-reactive protein (CRP) in subjects with elevated variables. There was also a slight reduction in body weight (BW) and body fat (BF), but these findings were not significant. In addition, the intake of honey allowed for a significant reduction in TG and CRP levels in subjects with high baseline values while it non-significantly reduced total cholesterol, LDL, TG, and CRP levels and increased HDL cholesterol in subjects with normal baseline values. In another study carried out on a group of obese prepubertal girls (10 ± 0.34 y) who had a dietary treatment, the intake of 15 g/day of wild flowers-forest-thyme honey (experimental group) or jam (control) showed similar results [16]. The decrease in BMI was greater in the experimental group but not in a significant way. HDL levels increased in the experimental group while it decreased in the control group. TG decreased in both groups which is greater in the experimental group; however, this difference was not significant. Raatz et al. [17] also found no significant differences in a trial conducted on overweight/obese subjects aged 35–55 years who had normal or impaired glucose tolerance. Subjects were supplemented with 50 g of Dutch Gold Honey (honey from different floral sources and geographic origin), sucrose, or corn syrup for 2 weeks. No changes in BW were observed throughout the trial, or in glucose and insulin concentrations during the glucose tolerance test. Systolic blood pressure (SBP) was unchanged while diastolic blood pressure (DBP) was significantly reduced between pre- and post-treatment in subjects supplemented with sucrose or syrup, indifferently of their glycaemic status. No significant changes in cholesterol levels were observed in either group, but TG levels increased significantly between pre- and post-treatment in sucrose-supplemented subjects. These results suggest that doses of 15 and 50 g/day are insufficient to produce significant improvements in cardiovascular risk factors. On the other hand, the intervention seems to be more effective in subjects with altered baseline values.

#### 3.1.3. Diabetic Subjects

Wahab et al. [18] carried out a study on healthy and diabetic post-menopausal women that showed that the intake of 20 g/day of Tualang sterilized honey supplied by Federal Agricultural Marketing Authorities (FAMA) (Malaysia) for 12 months had significant effects on lowering DBP and FBG. However, it had no significant effects on blood lipid profile, BMI, body composition, and waist circumference. In another study performed with type II diabetic subjects (57.2 ± 8.4 years), the experimental group was supplemented with increasing doses of Iranian natural unprocessed honey collected from Samans kandeh, Neka, Sari City, for 8 weeks, starting with 1.0 g/kg/day and increasing by 0.5 g/kg/day every 2 weeks until reaching 2.5 g/kg/day; while the control group was not supplemented with any substance. After 8 weeks of honey consumption, there were significant reductions in BW, TC, LDL, and TG, as well as a significant increase in HDL levels. There was also a decrease in FBG levels, although this was not significant. However, glycosylated hemoglobin (HbA1c) levels increased significantly in the honey-consuming group [19].

Mamdouh et al. [20] conducted a randomized crossover study on type I diabetic children. In the intervention period, the children were supplemented with 0.5 mL/kg/day of non-heated and non-irradiated Egyptian clover honey supplied by a beekeeper for 12 weeks. In the first period, statistically significant decreases in subscapular skinfold, FBG, post-prandial serum glucose, TC, HDL, and TG along with significant increases in fasting C-peptide and post-prandial C-peptide were observed in the intervention group compared to baseline. In the control group, no significant differences in any of the aforementioned parameters were observed. In the second period, significant reductions in midarm circumference, triceps skinfold, and fasting C-peptide were observed in the control group, while TC and LDL cholesterol increased significantly. On the contrary, significant decreases were observed in BMI, triceps skinfold, fasting serum glucose, HbA1C, total and LDL cholesterol, and TG in the intervention group. In addition, there were significant increases in fasting as well as post-prandial C-peptide and HDL.

Similarly, longer interventions with larger amounts of honey seem to have a greater effect on factors related to heart and vascular health in diabetic subjects.

#### 3.1.4. Subjects with Hyperlipidemia

Al-Waili NS et al. [21] conducted a study on a group of healthy subjects (25–48 years) and a group of patients with hypercholesterolaemia or hypertriglyceridaemia (35–55 years). Consumption of 75 g of natural honey for 15 days significantly reduced total cholesterol and CRP levels in patients with hyperlipidemia, as well as LDL levels but not significantly. However, the reduction in TC, LDL, TG, CRP, homocysteine, and FBG levels was not significant in healthy subjects. The control groups were supplemented with 75 g of artificial honey (honey–glucose mixture), which caused an increase in total and LDL cholesterol and CRP levels [21]. These results do not agree with those obtained by Munsted K et al. [22], who showed that consumption of the same amount of honey during the same period in subjects with hypercholesterolemia aged 35–87 years reduced TC and HDL levels, and increased LDL and TG levels. However, when differentiating between both sexes, it was observed that the LDL value increased in women in the control group supplemented with a sugar solution, but not in those supplemented with honey.

### 3.2. Glucose Tolerance

Table 2 shows glucose tolerance compared with honey intake versus other sugar solutions, using an oral glucose tolerance test.

#### 3.2.1. Healthy Subjects

A study on healthy men showed significantly lower increases in the concentration-time curve (AUC) profiles for glucose and a lower increase in plasma insulin after consumption of basswood (linden) honey compared to the other sugar solutions [23]. They have also documented significantly lower increases in plasma insulin and C-peptide after consumption of natural or clover honey versus other sugar solutions in healthy subjects [21].

#### 3.2.2. Diabetic Subjects

Significantly lower increases in blood glucose levels have been observed in subjects with type II diabetes after ingestion of natural honey [21] and clover honey [21,24] compared to the other sugar solutions. A study carried out with healthy and type I diabetic subjects also showed a significantly lower increase in blood glucose levels following Egyptian clover honey consumption, and a significantly higher increase in C-peptide levels [25].

These results indicate that replacing sugar with honey could be beneficial for both healthy and diabetic patients.

**Table 2:** Effects of honey on glucose tolerance.

Honey	Dose	Test Duration	Subjects	Physiological Parameter	Effect	References
Basswood (linden) honey	75 g vs. glucose-fructose	120 min	Healthy men 27.7 years	Increase in BGL	↓	[23]
				AUC for glucose	↓ ^a^	
				Increase in BIL	↓ ^a^	
				Increase in C-peptide	↓	
Natural honey	75 g vs. dextrose	180 min	Healthy subjects 25–42 years	Increase in BGL	↓	[21]
				Increase in BIL	↓ ^a^	
				Increase in C-peptide	↓ ^a^	
	70 g vs. glucose		Type II diabetic patients	Increase in BGL	↓ ^a^	
Sue Bee honey (clover honey) 100% pure	75 g honey vs. glucose	120 min	Type II diabetic patients 50 ± 9.7 years	Increase in BGL	↓ ^a^	[24]
Unprocessed Egyptian clover honey supplied by a beekeeper	2.3 g/kg	120 min	Healthy subjects and type I diabetic patients 10.02 years	Increase in BGL	↓ ^a^	[25]
				Increase in C-peptide	↑ ^a^	

BGL: blood glucose level; BIL: blood insulin level; AUC: areas under the concentration–time curve; ↑: increase; ↓: decrease. ^a^ Significantly different (*p* < 0.05) from the control groups.

### 3.3. Appetite and Food Intake

The results of studies on the effects of honey consumption on appetite and energy intake are shown in Table 3.

#### 3.3.1. Healthy Subjects

In a study conducted by Al-Tamimi et al. [14] on healthy subjects aged 24–57 years, the intake of 1.5 g/kg/day of a mixture of four types of clover honey promoted a significantly lower intake of energy, carbohydrates, and sugars compared to the sucrose-supplemented group. The inclusion of 42.7 g of pure clover honey in a 440 kcal meal showed a significant reduction in post-prandial blood glucose while lactate increased in healthy women aged 18–40 years that consumed the honey meal versus those who received a meal including 35.5 g of sucrose [26]. A similar pattern was observed for insulin, but the effect was not significant. The decrease in ghrelin, however, showed no significant difference between the two meals. Both post-prandial peptide YY and leptin levels did not change significantly over time and did not differ according to treatment; however, the AUC for peptide YY was significantly higher after the honey meal. Hunger and satiety were assessed, and participants received a free-choice meal 240 min after the test meal was consumed. Post-prandial satiety scores were significantly higher after the honey meal versus the sucrose one at 60 min. It could be that clover honey is satiating. In addition, the AUC for hunger during the 240 min following ingestion of the test meal tended to be lower with the honey meal than with the sucrose meal. Regarding meal-induced thermogenesis, there were no significant differences between the two groups and the same trend was found for energy and macronutrient intake in the free-choice meal between the two groups [26].

#### 3.3.2. Diabetic Subjects

Contrarily, although the trend appears to be positive in healthy subjects, Bahrami et al. [19] reported no significant differences in diabetic patients after supplementation with Iranian natural honey for 8 weeks in energy intake or protein, fat, carbohydrate or sugar intake.

### 3.4. Alcohol Metabolism

In terms of the effect of honey on alcohol metabolism (Table 4), the inclusion of freshly harvested Nigerian citrus (Citrus sinensis Osbeck) honey from the delta region of the River Niger along with alcohol intake has shown a significant decrease in the degree and time of intoxication in healthy adults [27,28]. Therefore, honey could be a promising anti-intoxication agent. However, in men, the consumption of alcohol and honey seems to cause a significant increase in TG levels [28]. As a result, further studies would be necessary to establish recommendations.

### 3.5. Cancer

Table 5 displays different studies conducted on cancer patients to test the effect of honey intake on various cancer-related complications, such as mucositis, weight loss, and xerostomia. In this regard, mouth washing with a solution of natural Baran-Baghro honey from Iran in water (1:20, *v/v*) for 4 weeks significantly reduced the severity of mucositis in adult patients with myeloid leukemia undergoing chemotherapy and significantly increased the patients’ body weight [29]. The same results were shown in another study carried out on patients with head and neck cancer receiving radiotherapy, who were treated with mouthwashes of a solution of pure and filtered thyme honey in water (1:5, *v/v*) for 6 months. In this case, it significantly reduced the severity of mucositis and weight loss, and significantly increased overall health and quality of life in the experimental group [30]. In agreement, Khanal et al. [31] reported a significant reduction in mucositis in adult patients with oral carcinoma, the mouth rinse was performed with 20 mL of Western Ghats Forest honey for 6 weeks. In other studies, involving adult patients with head and neck cancer receiving chemo-radiotherapy, the treatment consisted of smearing 20 mL of honey all over the mouth and swallowing it slowly. Treatments with clover honey, thyme, and astragalus honey or tea plant honey significantly reduced the severity of mucositis [32,33,34]. In addition, thyme and astragalus honey and tea plant honey significantly reduced weight loss in cancer patients [33,34], and clover honey significantly reduced Candida colonization, which is the most common clinical infection of the oropharynx in patients receiving radiotherapy [32]. In another study carried out with patients with head and neck cancer aged 61 years on average who are receiving radiotherapy, chemotherapy or surgery, patients in the experimental group were given a solution of pure filtered thyme honey in water (1:5, *v/v*) to swish around in their mouth and swallow slowly. There were significant reductions in the level of xerostomia, pain, and dysphagia compared to the control group as well as a significant increase in patients’ quality of life measured by a Quality of Life scales containing 15 items (Dirix XQ) [35]. However, the same treatment with Manuka honey in the same type of patients did not produce significant changes in the severity [36,37] or duration of mucositis [37]. Moreover, the Manuka honey was not well tolerated by patients. Studies in pediatric patients also showed a significant reduction in the severity of mucositis using treatments with Egyptian clover honey [38] or Turkish flower honey [39].

A study conducted on adult cancer patients with neutropenia showed a significant improvement in neutrophil levels when supplementing these patients with 5 g/day of Life-Mel honey for 5 days [40]. Similarly, a significant reduction in febrile neutropenia episodes with the supplementation of 2.5 g/kg twice a week of Egyptian clover honey in a study in pediatric patients was observed. In addition, the intervention group significantly improved their hemoglobin levels compared to the control group [41].

Forest, thyme, clover, and tea tree honeys appear to be very effective in improving mucositis symptoms in cancer patients undergoing radiotherapy and/or chemotherapy; however, Manuka honey does not produce improvements and is not well tolerated by these patients; therefore, it does not appear to be recommended.

**Table 5:** Effects of honey on cancer patients.

Honey	Dose	Duration	Subjects	Physiological Parameter	Effect	Reference
Natural Baran-Baghro honey from Iran	1:20 honey:water Mouthwash	4 w	Acute myeloid leukemia patients receiving chemotherapy >18 years	Mucositis severity	↓ *^,a^	[29]
				Body weight	↑ *^,a^	
Pure and filtered thyme honey	1:5 honey:water Mouthwash	6 m	Head and neck cancer patients receiving radiotherapy 61.53 years	Mucositis severity	↓ *^,a^	[30]
				Weight loss	↓ ^a^	
				Global health	↑ *^,a^	
				Life quality	↑ *^,a^	
Western Ghats forests honey	20 mL Mouthwash	6 w	Oral carcinoma patients receiving radiotherapy >18 years	Mucositis severity	↓ ^a^	[31]
Pure and filtered natural clover honey	20 mL pure honey Rinse + swallow	7 w	Head and neck cancer patients receiving chemotherapy 48.20 ± 15.63 years	Mucositis severity	↓ ^a^	[32]
				Candida colonization	↓ ^a^	
Pure natural honey from Thymus and Astragale in the Albroz mountains in northern Iran	20 mL pure honey Rinse + swallow	6 w	Head and neck cancer patients receiving radiotherapy 57.0 ± 12.0 years	Mucositis severity	↓ *^,a^	[33]
				Weight loss	↓ ^a^	
Tea plant honey from Cameron Highland of peninsular Malaysia	20 mL pure honey Rinse + swallow	7 w	Head and neck cancer patients receiving radiotherapy 14–89 years	Mucositis severity	↓ ^a^	[34]
				Body weight	↑ ^a^	
Pure and filtered thyme honey	1:5 honey:water Rinse + swallow	6 w	Head and neck cancer patients receiving radiotherapy or chemotherapy or surgery 61.53 ± 13.50 years	Xerostomia level	↓ ^a^	[35]
				Quality life	↑ ^a^	
				Pain	↓ ^a^	
				Dysphagia	↓ ^a^	
Irradiated organic manuka honey	5 mL Rinse + swallow	6 w	Head and neck cancer patients receiving radiotherapy	Mucositis severity	↓	[36]
Active manuka honey	20 mL (98% honey) Rinse + swallow	6 w	Head and neck cancer patients receiving radiotherapy 38–85 years	Incidence of servere mucositis	↑	[37]
				Mucositis severity	↓	
				Mucositis duration	↓	
Egyptian clover honey from El Mahala, Gharbia Governorate	0.5 g/kg/d Rinse + swallow	10 d	Lymphoblastic leukaemia patients receiving chemotherapy 6.9 ± 3.8 years	Mucositis recovery time	↓ ^a^	[38]
Turkish Flower honey from the highlands of Zonguldak Province, in the Western Black Sea Region of Turkey	3.70–30.96 g Rinse + swallow	21 d	Children treated in a paediatric intensive care unit (PICU) 7.25 years	Mucositis severity	↓ *^,a^	[39]
Life-Mel honey from Express Honey, Tzuf Globus, Israel	5 g/d	5 d	Cancer patients with neutropenia 57 years	Neutrophil level	↑ *	[40]
				Haemoglobin level	↑	
				Thrombocytes level	↑	
Egyptian unprocessed clover honey collected from Al Mahala-Gharbia Governorate	2.5 g/kg twice weekly	Crossover Two 12 w periods	Children with acute lymphoblastic leukemia 5.4 ± 2.4 years	Febrile neutropenia episodes	↓ ^a^	[41]
				Number of patients admitted in hospital	↓	
				Duration of hospital stay	↓	
				Haemoglobin level	↑ ^a^	

d: day; w: week; ↑: increase; ↓: decrease. * Significant differences (*p* < 0.05) within group between baseline and the end. ^a^ Significantly different (*p* < 0.05) from the control group.

### 3.6. Cough and Gastroenteritis in Infants

Table 6 shows different studies in pediatric patients with common cold or URTIs. Several studies have linked the consumption of different types of honey (Buckwheat honey, Iranian, eucalyptus, citrus, Labiatae and Nairobi dark honey) with significant reductions in frequency [42,43,44,45,46], bothersome [44,45,46], and severity [43,44,45] of nocturnal cough, as well as in the combined symptom score of URTIs [42,44,45,46]. Significant improvements in sleep quality have also been observed in children and parents [43,44,45,46]. Contrarily, a study in which children were supplemented with acacia honey for 2 days showed no significant differences with honey consumption versus placebo [47]. On the other hand, an early study carried out on children with gastroenteritis showed a significant reduction in recovery time from bacterial gastroenteritis by substituting pure honey for glucose in the oral rehydration solution [48].

### 3.7. Antimicrobial and Wound Healing Effects

Studies on the antimicrobial and wound healing effects of honey are described in Table 7. Rinses with a solution of multifloral processed honey in water (1:1, *v/v*) for 5 days significantly reduced dental plaque in healthy subjects, although a 0.2% chlorhexidine solution was found to be more effective [49]. Banaeian et al. [50] studied the influence of Iranian honey on vulvovaginal candidiasis, and they found significant reductions in inflammation, discharge, and itching after 8 days of treatment with a 70% honey cream. They concluded that although treatment with 1% clotrimazole was more effective, honey could be an alternative for the treatment of vulvovaginal candidiasis due to its wide availability and cost-effectiveness [50]. In a study conducted on children with pyomyositis abscesses, gauze soaked in natural raw honey, or a medical solution was applied to the wounds for 21 days. Honey significantly improved wound healing and reduced the duration of hospital stay [51]. Similarly, Lavaf et al. [52] demonstrated that a 30% Iranian honey cream significantly increased healing and reduced discharge from episiotomy wounds in nulliparous women. Several studies have tested the effect of honey dressings on patients with diabetic foot ulcers. Muhammad Imran et al. [53] in their study using Beri honey observed significant improvements in both wound healing and healing time in the experimental group compared to the control group treated with normal saline dressing. Similar results were reported by Moghazy et al. [54] who observed a significant improvement in healing and healing time of diabetic foot ulcers after treatment with pure Egyptian honey dressings; however, no control group was used in this study. On the contrary, Shukrimi et al. [55] observed no significant effect with clean non-sterile honey treatment compared to povidone iodine.

Although honeys from different origins have been shown to have antimicrobial effects, they are not superior to pharmacological treatments, such as chlorhexidine, clotrimazole or povidone-iodine. However, due to their low cost, wide availability, and lack of side effects, honey could be an alternative to conventional treatments, although more research is needed.

**Table 6:** Effects of honey on cough and gastroenteritis in children.

Honey	Dose	Duration	Subjects	Physiological Parameter	Effect	References
Buckwheat honey	Children aged 2 to 5 (1/2 teaspoon), 6 to 11 (1 teaspoon), 12 to 18 (2 teaspoons) Single dose	1 d	Children with upper URTIs 5.02 ± 3.99 years	Cough frequency	↓ ^a^	[42]
				Combined symptom score	↓ ^a^	
				Bothersome cough	↓	
				Cough severity	↓	
				Sleep quality	↑	
				Parents’ sleep quality	↑	
Iranian natural honey from Kafi-Abad, Yazd	2.5 mL Single dose	1 d	Children with URTIs 3.15 ± 0.93 years	Cough frequency	↓ ^a^	[43]
				Cough severity	↓^a^	
				Sleep quality	↑ ^a^	
				Parents’ sleep quality	↑ ^a^	
Eucalyptus, citrus or Labiatae honey	10 g Single dose	1 d	Children with URTIs 2.4 years	Cough frequency	↓ ^a^	[44]
				Combined symptom score	↓ ^a^	
				Bothersome cough	↓ ^a^	
				Cough severity	↓ ^a^	
				Sleep quality	↑ ^a^	
				Parents’ sleep quality	↑ ^a^	
Nairobi dark honey	Children aged 1 to 2 (2.5 mL), 2 to 6 (5 mL), 6 to 12 (7.5 mL) Three times daily	5 d	Children with a common cold 1–12 years	Cough frequency	↓ ^a^	[45]
				Combined symptom score	↓ ^a^	
				Bothersome cough	↓ ^a^	
				Cough severity	↓ ^a^	
				Cough duration	↓ ^a^	
				Sleep quality	↑ ^a^	
				Parents’ sleep quality	↑ ^a^	
Two kinds of Iranian honey: Kimia honey and Golha honey	Children aged 1 to 6 (2.5 mL), 7 to 12 (5 mL), Two doses	2 d	Children with URTIs 3.5 ± 1.6 years	Cough frequency	↓ ^b^	[46]
				Combined standard score	↓ ^a^	
				Bothersome cough	↓ ^a^	
				Sleep quality	↑ ^b^	
				Parents’ sleep quality	↑ ^a^	
Acacia honey	3 mL Single dose	2 d	Children with URTIs 2.5 years	Cough frequency	↓	[47]
				Combined symptom score	↓	
				Bothersome cough	↓	
				Cough severity	↓	
				Cough duration	↑	
				Sleep quality	↑	
Pure honey	50 mL/L of rehydration solution vs. 50 mL/L of glucose	Duration of gastroenteritis	Children with gastroenteritis 1.39 ± 1.82 years	Bacterial gastroenteritis recovery time	↓ ^a^	[48]

d: day; URTIs: upper respiratory tract infections; ↑: increase; ↓: decrease. ^a^ Significantly different (*p* < 0.05) from the control group. ^b^ Significantly different (*p* < 0.05) for one type of honey.

**Table 7:** Effects of honey on wounds.

Honey	Dose	Duration	Subjects	Physiological Parameter	Effect	References
Multifloral processed honey	1:1 honey:water Mouthrinse 10 mL twice a day	5 d	Healthy subjects 20–24 years	Tooth plaque	↓ *	[49]
Iranian honey from Chaharmahal and Bakhtiari region	70:30 honey:neutral cream 5 g/d	7 d	Women with vulvovaginal candidiasis 34.3 ± 8.6 years	Inflammation	↓ *	[50]
				Discharge	↓ *	
				Itching	↓ *	
Natural raw honey	Honey-soaked gauze vs. medical solution	21 d	Children with pyomyositis abcesses 4.5 ± 4.0 years	Wound healing	↑ ^a^	[51]
				Duration of hospital stay	↓ ^a^	
Iranian honey from Qamsar region	30% honey cream 1 knuckle/d	14 d	Nulliparous women with episiotomy wound 24.7 ± 4.0 years	Discharge	↓ ^a^	[52]
				Wound healing	↑ ^a^	
				Pain	↓	
Beri-irradiated honey collected from Karak, Pakistan	Once or twice daily or every 48 h	4 m	Patients with diabetic foot ulcers 54 years	Wound healing	↑ ^a^	[53]
				Wound healing time	↓ ^a^	
Pure raw untreated clover honey supplied by the Firm of Faculty of Agriculture, Alexandria University	Honey-soaked gauze	3 m	Patients with diabetic foot ulcers 52.3 years	Ulcer size	↓ *	[54]
				Ulcer grade	↓ *	
				Ulcer stage	↓ *	
				Inflammation	↓ *	
				Discharge	↓ *	
				Wound healing	↑ *	
Clean non-sterile pure honey packed by Barnes for Honey Cooperation of Australia	Once daily	36 d	Patients with diabetic foot ulcers 31–51 years	Wound healing time	↓	[55]
				Discharge	↓	
				Edema	↓	

d: day; m: month; ↑: increase; ↓: decrease. * Significant differences (*p* < 0.05) within group between baseline and the end. ^a^ Significantly different (*p* < 0.05) from the control group.

### 3.8. Other Effects

Table 8 shows different effects of honey not discussed in the previous sections. A study carried out on healthy subjects showed that both low- and high-antioxidant buckwheat honey significantly increased plasma total phenolic concentration 2 h after consumption. However, this effect extended to 6 h only after consumption of high-antioxidant buckwheat honey. The same effect was observed on total plasma antioxidant capacity after consumption of these two honeys. Both honeys also increased total plasma reducing capacity 2 and 6 h after consumption [56]. A study on subjects with blepharitis showed a significant improvement in dryness of the eye, tear film quality, and ocular surface, as well as a decrease in microbial colonization in patients treated with a Manuka honey microemulsion cream for 3 months [57]. In contrast, the inclusion of 1 tablespoonful/d of natural Bristol honey or processed honey showed no improvement over placebo in the symptoms of allergic rhinoconjunctivitis [58]. Wallace et al. [59] found no significant change in IgE levels after consumption of 20 g of multifloral honey or manuka honey UMF 20+ for 4 weeks in healthy adults. There was also no effect on the number of intestinal bacteria of the *Bacteroides*, *Bifidobacterium*, *Lactobacillus*, *Escherichia coli,* and *Clostridium* groups. Farahani et al. [60] also observed no effect of Astragalus honey consumption on symptoms of dysmenorrhoea in female students with an average age of 22.

## 4. Discussion

According to the results of the present review, it appears that honey from clover, basswood, citrus, thyme, tea plant, flowers, buckwheat, eucalyptus, Labiatae, and Manuka has beneficial effects on certain parameters, such as cardiovascular risk factors, satiety, glucose tolerance, mucositis symptoms in cancer patients, URTIs symptoms in children, wound healing, etc.

It has been demonstrated that honey consumption can influence plasma lipid, glucose, and insulin levels through different biochemical mechanisms. The decrease in blood glucose may be due to the fact that honey has a stimulatory effect on insulin secretion and improves insulin sensitivity [13]. Honey also increases the production of hydrogen peroxide, which has similar effects to insulin [61]. In addition, it is possible that honey consumption stimulates nitric oxide synthase [62] and the increase in nitric oxide (NO), in turn, stimulates insulin release [63] since it contains NO metabolites. It has also been reported that honey consumption decreases plasma levels of some prostaglandins [62,64] that inhibit insulin secretion [13], constituting another pathway of increased insulin release. Moreover, honey contains zinc and copper, which play an important role in insulin and glucose metabolism [21,65]. The high fructose content of honey may also decrease the hyperglycemic glucose response by stimulating glucokinase to deliver glucose to the liver [15]. However, long-term glucose consumption can have negative effects on digestion, absorption, hormone levels, appetite, and liver metabolism, which can lead to the development of insulin resistance, obesity, and cardiovascular disease [66]. These negative effects have not been observed with honey consumption; therefore, it is believed that other components of honey, such as antioxidants (e.g., phenolic compounds and some vitamins), may contribute to the reduction in the negative effects produced by fructose consumption [19]. In addition, some characteristic flavonoids of honey, i.e., apigenin, luteolin, galangin-3-methyl ether, kaempferol, naringenin, rutin, quercetin, and myricetin have shown significant reductions in blood glucose levels and beneficial effects on dyslipidemia in animals [67,68,69,70]. This may be due to the inhibitory effect of flavonoids on mammalian alpha-amylase [71,72,73], which catalyzes the hydrolysis of the alpha-glycosidic bonds of high molecular weight polysaccharides releasing glucose and maltose. In addition, available data show that phenolic compounds from honey are bioavailable and increase the antioxidant activity of plasma [42,44]. The antioxidant activity of the phenolic compounds is attributed to their capacity to eliminate free radicals by donating hydrogen atoms, electrons or metallic cations, due to their structure (number and positions of the hydroxyl groups and the nature of the substitutions in the aromatic rings) and due to their binding to organic acids and sugars [1]. On the other hand, phenolic compounds promote the maintenance and recovery of the balance of the intestinal microbiota since they can stimulate the secretion of antioxidant enzymes, such as superoxide dismutase (SOD), catalase (CAT), glutathione peroxidase (GPx), glutathione reductase (GR), and peroxiredoxins that block reactive oxygen species (ROS) or stimulate endogenous defense system [1].

Its content of antioxidants, such as beta-carotene, vitamin C, and uric acid and its mineral content, such as copper, manganese, selenium, and zinc may also be responsible for the effects on blood lipids [19]. These elements may increase the catabolism of fats, leading to a decrease in serum lipid levels [19]. In addition, these antioxidants decrease oxidized LDL [13]. Among vitamins, honey contains niacin, which strongly inhibits lipolysis in adipose tissue, leading to a decrease in hepatic TG synthesis, and thus plasma TG levels [13]. TG synthesis is necessary for the synthesis of VLDL from which LDL in blood plasma is derived. It is therefore believed that niacin can also lower plasma LDL and total cholesterol levels [13]. On the other hand, insulin can stimulate protein lipase, increasing lipid metabolism, which results in a decrease in serum lipid levels [13,74]. The increase in HDL associated with honey consumption may be due to the fact that HDLs obtain cholesterol from cell membranes and other lipoproteins, such as LDL and transport it to the liver. As honey consumption decreases LDL, less HDL would be used to transport cholesterol to the liver, which may increase serum HDL levels [13], although further studies are needed to confirm this mechanism. In addition, the niacin content of honey may be responsible for the increase in HDL levels [13]. Concerning phenolic compounds, the cardioprotective effect of flavonoids has been widely demonstrated through the reduction in blood platelet activity, the prevention of LDL oxidation, and the improvement of coronary vasodilation [7].

Plasma concentrations of C-peptide effectively reflect endogenous insulin secretion; therefore, it is considered a good marker of insulin secretion. Increases in C-peptide levels following honey consumption in both healthy and diabetic subjects demonstrate that honey may stimulate both healthy and diseased pancreatic beta cells. It is thought that due to its anti-inflammatory [62], antioxidant [75], antiviral and probiotic [76] properties, honey may contribute to the healing of diseased beta cells [25]. On the other hand, Panero et al. [77] observed that higher levels of C-peptide in type I diabetic patients confer a statistically significant protective effect against the development of microvascular complications. Furthermore, due to the *Lactobacillus* and *Bifidobacterium* involved in its production, honey is considered a fermented and, consequently, probiotic product, which could reduce inflammation and intestinal permeability, and change the composition of the intestinal flora, these factors being implicated in the pathogenesis of type I diabetes mellitus [78]. Moreover, the antidiabetic and hypoglycemic capacity of honey can be attributed to its antioxidant ability (thanks to its phenolic compound content), as the pathogenesis of diabetes mellitus appears to be closely associated with the presence of oxidative stress and ROS [7].

On the other hand, although the role of honey in weight loss is still unclear and more studies are needed to clarify how honey consumption may affect body composition, honey consumption has been associated with increased serum levels of antioxidants, such as vitamin C, β-carotene, uric acid and glutathione reductase, and the total phenolic content which results from phenolic antioxidants in the honey [56]. These compounds appear to increase diet-induced thermogenesis, and thus may be related to the weight loss associated with honey consumption versus other sweeteners [79].

Despite the fact that the mechanism of action of honey on oral mucositis is not well established, it may be due to the analgesic [80], antimicrobial [81], and wound-healing [82] properties that have been attributed to this food. In addition, qualities, such as high viscosity, high osmolarity, and low pH level of honey enhance its bacterial and fungal inhibitory activity [80]. On the one hand, since honey contains a characteristic sweetness [80] as well as ascorbic, citric, and malic acids [35], it increases salivation, and thus promotes repair and healing of the oral mucosa. On the other hand, weight loss is very common in cancer patients and has negative effects, such as increased risk of infection and reduced quality of life, treatment responses, and survival [83]. The positive effect of honey on body weight in cancer patients may be due to its positive effects on mucositis, as this results in increased oral food intake [84]. The positive effect of honey against neutropenia could be associated with its antioxidant [40], antimicrobial, and immunomodulatory [41] properties. Another hypothesis is that honey could increase levels of granulocyte colony stimulating factor (G-CSF) [41], which induces neutrophil production [85] by increasing TNF-α and IL-1. The evidence seems to indicate that honey is quite beneficial for cancer patients, although more standardized and longer-term studies are needed to confirm these effects.

The positive effect of honey on cough may be due to its antioxidant, antimicrobial [42,43], anti-inflammatory, and antiviral effects [46]. In addition, some of the studies used dark honey [42,44], which tends to have a higher content of phenolic compounds that have been related to the antioxidant properties of honey [42,46], and thus may have contributed to the positive effects observed. As honey is a sweet substance that increases salivation and mucus secretion from the respiratory tract, it may have a demulcent effect on the pharynx and larynx, and thus reduces dry and unproductive cough [86]. In addition, these secretions can improve mucociliary clearance in the airways through expectoration [86]. On the other hand, the anatomical relationship between the nerve fibers that initiate coughing and the nerve fibers that taste sweetness may lead to an interaction between the two and favor antitussive effects [86]. Therefore, there appears to be benefits with the addition of honey to conventional treatment. It is worth mentioning that children under 1 year of age should not consume honey due to the high risk of developing botulism [87,88].

Table 9 presents a summary of the most significant effects of honey on human health found in this review. Doses of 70 g/d seem to have beneficial effects on cardiovascular risk factors in healthy subjects as well as in hyperlipidemic subjects; however, beneficial effects have been observed with doses of 20 g/d and above in diabetic subjects. In addition, glucose tolerance is better after consumption of honey than other sugar solutions in both diabetic and healthy subjects. Supplementation with clover honey has been shown to have satiating effects. Doses of 1–1.25 g/kg of honey reduce intoxication time after alcohol ingestion. In patients with leukemia and head and neck cancer, improvements in mucositis and decreases in weight loss have been observed when using solutions of different types of honey (forest, thyme, clover, and tea tree honey) for rinses or mouthwashes with ingestion. However, Manuka honey does not seem to be recommended for these patients. Improvements in URTI symptoms have also been observed in children after ingestion of 2.5–10 g of different types of honey. There also seems to be an acceleration in wound healing and a decrease in wound secretion with the application of honey creams in pyomyositis abscesses in children, episiotomy wounds, and diabetic foot ulcers, although these effects do not outweigh conventional treatments.

Based on the available data, the average ingestion dose used is estimated to be 40.71 ± 30.59 g/day or 1.38 ± 0.59 g/kg/day, and the topical dose 12.50 ± 10.61 g, with the average duration of the studies being 8.5 ± 8.9 weeks. It should be noted that the included studies involve small samples, lack a standardized protocol, and differ in types of honey, doses used, duration of interventions and population groups (e.g., healthy subjects, overweight or obese subjects, diabetics, cancer patients, etc.), which makes it difficult to compare results and establish specific recommendations. Therefore, despite the promising positive effects of honey intake (Table 9), more evidence including the same type of honey, dosage, and trial protocols is necessary to establish a real cause and effect relationship between honey intake and the described healthy effects. In fact, as early as 2010, the European Food Safety Authority (EFSA) issued a report concluding that honey was not sufficiently characterized in relation to the claimed effects, i.e., “respiratory health through presence of antioxidant phytochemicals”, “the unique composition and ratio of effective substances adds energy to the human body”, and “it stimulates the whole metabolism and the immune system” [89].

## 5. Conclusions

To date, a limited number of studies have been carried out. Along with a lack of standardized research, the variety of methodology used, as well as differences in the duration of the interventions, the age of the subjects, and their physiological or pathological conditions is difficult to compare between them. The type of honey and the doses used in the different studies also vary widely, which does not allow the beneficial effects to be attributed to a specific honey nor a specific dose. However, despite being halfway between consideration as a functional food or a harmful food due to its high sugar content, more beneficial effects of honey intake have been observed than no or negative effects, especially when its intake replaces the intake of other sweeteners. The main beneficial effects have been observed on cardiovascular health in healthy, diabetic, and hyperlipidaemic subjects on glucose tolerance in healthy and diabetic subjects, on mucositis in cancer patients, on URTIs in children, and on wound healing. Therefore, honey could be a safe adjuvant to be administered to people aged more than 1 year old alongside drugs currently used for certain diseases. However, it should not be forgotten that honey is a high sugar food, and it should be consumed occasionally and with moderation. More studies are necessary to establish more specific recommendations on honey consumption.

## Figures and Tables

**Table 1:** Effects of honey on cardiovascular risk factors.

Honey	Dose	Duration	Subjects	Physiological Parameter	Effect	Reference
Natural honey	70 g/d vs. sucrose	6 w	Healthy males 25.51 ± 1.63 years	TG	↓ *^,a^	[12]
				TC	↓ *^,a^	
				LDL	↓ *^,a^	
				HDL	↑ *^,a^	
Natural, unprocessed honey purchased from Ilyas Traders, Charsadda, Khyber Pakhtunkhwa, Pakistan	Diet + 70 g/d vs. diet	1 m	Healthy Pakistani males 20.13 ± 0.14 years	Increase in FBG	↓ ^a^	[13]
				TG	↓ ^a^	
				TC	↓ *^,a^	
				LDL	↓ *^,a^	
				HDL	↑ *^,a^	
Mixture of four types of clover honey obtained from Golden Heritage Foods, Smitty Bee Honey, Millers Honey Company, and Marshall’s Farm Natural Honey	1.5 g/kg/d honey vs. sucrose	1 m	Healthy subjects 32.9 ± 1.7 years	FBI	=	[14]
				TC	↓	
				LDL	↓	
				HDL	↓	
Iranian natural honey	70 g/d vs. sucrose	1 m	Overweight or obese subjects 42.6 ± 8.6 years	TG	↓ ^b^	[15]
				TC	↓	
				LDL	↓	
				HDL	↑	
				FBG	↓ *	
				CRP	↓ ^b^	
				BW	↓	
				BF	↓	
				BMI	↓ *	
Wild flowers-forest-thyme honey produced by Attiki	Diet + 15 g/d vs. diet + marmelade	6 m	Obese girls 10.55 ± 0.34 years	BMI	↓	[16]
				TG	↓	
				TC	↑	
				LDL	↑	
				HDL	↑	
				OGT	↑	
Dutch Gold Honey (honey from different floral sources and geographic origin)	50 g/d vs. sucrose or corn syrup	2 w	Glucose-tolerant with overweight or obesity 38.9 ± 3.6 years	BW	↑	
				BMI	=	[17]
				SBP	=	
				DBP	=	
				TG	↑ *	
				TC	↑	
				LDL	=	
				HDL	=	
				FBG	↓	
				FBI	↓	
			Glucose-intolerant with overweight or obesity 52.1 ± 2.7 years	BW	↑	
				BMI	↑	
				SBP	↑	
				DBP	=	
				TG	↑	
				TC	↑	
				LDL	↑	
				HDL	↑	
				FBG	↑	
				FBI	↑	
Tualang sterilized honey supplied by Federal Agricultural Marketing Authorities (FAMA), Malaysia	20 g/d vs. honey cocktail	12 m	Post-menopausal healthy and diabetic women 58.1 ± 3.7 years	SBP	↓	[18]
				DBP	↓ ^a^	
				TC	↓	
				LDL	↑	
				HDL	↓	
				TG	↑	
				FBG	↓ ^a^	
				BMI	↑	
				BF	↑	
				WC	↑	
Iranian natural unprocessed honey collected from Samans kandeh, Neka, Sari City	1 g/kg/d first 2 w 1.5 g/kg/d second 2 w 2 g/kg/d third 2 w 2.5 g/kg/d last 2 w	8 w	Type II diabetes 57.2 ± 8.4 years	BW	↓ *^,a^	[19]
				FBG	↓	
				HbA1c	↑ *	
				TG	↓ *	
				TC	↓ *	
				LDL	↓ *	
				HDL	↑ *	
Unprocessed Egyptian clover honey supplied by a beekeeper	0.5 mL/kg/d	Crossover study Two 12 w intervention periods	Type I diabetes 4.7 ± 4.28 years	SSFT	↓ *	[20]
				MC	↓	
				TSFT	↓	
				FBG	↓ *	
				TG	↓ *	
				TC	↓ *	
				LDL	↓ *	
				HDL	↑ *	
				C-peptide	↑ *	
				HbA1C	↓ *	
Natural honey	75 g/d	15 d	Healthy subjects	FBG	↓	[21]
				TG	↓	
				TC	↓	
				LDL	↓	
				HDL	↑	
				CRP	↓	
			Patients with hyperlipidemia	TC	↓ *	
				LDL	↓	
				CRP	↓ *	
Mixed blossom honey from Europe, Central America, and South America	75 g/d vs. sugar solution	2 w	Subjects with hypercholesterolemia 35–86 years	TG	↑	[22]
				TC	↓	
				LDL	↑	
				HDL	↓	

d: day; w: week; m: month; TG: triglycerides; TC: total cholesterol; LDL: low-density lipoproteins; HDL: high-density lipoproteins; FBG: fasting blood glucose; FBI: fasting blood insulin; CRP: C-protein reactive; BW: body weight; BF: body fat; BMI: body mass index; OGT: oral glucose tolerance; SBP: systolic blood pressure; DBP: diastolic blood pressure; WC: waist circumference; HbA1c: glycosylated haemoglobin; SSFT: subscapular skin fold thickness; MC: midarm circumference; TSFT: triceps skin fold thickness; ↑: increase; ↓: decrease; =: unchanged. * Significant differences (*p* < 0.05) within group between baseline and the end. ^a^ Significantly different (*p* < 0.05) from the control groups. ^b^ Significantly different (*p* < 0.05) in subjects with elevated baseline variables.

**Table 3:** Effects of honey on appetite and food intake.

Honey	Dose	Duration	Subjects	Physiological Parameter	Effect	Reference
Mixture of four types of clover honey obtained from Golden Heritage Foods, Smitty Bee Honey, Millers Honey Company, and Marshall’s Farm Natural Honey	1.5 g/kg/d honey vs. sucrose	1 m	Healthy subjects 24–57 years	Increase in energy intake	↓ ^a^	[14]
				Increase in carbohydrate intake	↓ ^a^	
				Increase in sugar intake	↓ ^a^	
Pure clover honey	42.7 g vs. 35.5 g of sucrose	1 d	Healthy women 21.8 ± 2.9 years	Increase in post-prandial glucose	↓ ^a^	
				Increase in post-prandial insulin	↑	
				Post-prandial leptin	↑	[26]
				Post-prandial ghrelin	↓	
				Post-prandial peptide YY	↑	
				Hunger rate	↓	
				Satiety rate	↑ ^a^	
				Thermogenesis	↑	
				Energy intake	↑	
				Carbohydrate intake	↑	
				Sugar intake	↓	
Iranian natural unprocessed honey collected from Samans kandeh, Neka, Sari City	1 g/kg/d first 2 w 1.5 g/kg/d 2 w 2 g/kg/d 2 w 2.5 g/kg/d last 2 w	8 w	Type II diabetes 57.2 ± 8.4 years	Energy intake	↓	[19]
				Energy from protein	↓	
				Energy from carbohydrate	↑	
				Energy from fat	↓	
				Sugar intake	↑	

d: day; w: week; m: month; ↑: increase; ↓: decrease. ^a^ Significantly different (*p* < 0.05) from the control groups.

**Table 4:** Effects of honey on alcohol metabolism.

Honey	Dose	Duration	Subjects	Physiological Parameter	Effect	References
Freshly harvested Nigerian citrus (Citrus sinensis Osbeck) honey from the delta region of the River Niger	0.5 mL/kg of ethanol + 1 mL/kg of honey	1 d	Healthy subjects 25–35 years	Blood alcohol clearance rate	↓ *	[27]
				Intoxication time	↓ *	
				Intoxication degree	↓	
Freshly harvested Nigerian citrus (Citrus sinensis Osbeck) honey from the delta region of the River Niger	0.5 g/kg of ethanol + 1.25 mL/kg of honey	1 d	Healthy men 23.6 ± 7.4 years	Intoxication time	↓ *	[28]
				Intoxication degree	↓ *	
				TG	↑ *	
				Blood pressure	↑	

d: day; TG: Triglycerides; ↑: increase; ↓: decrease. * Significant differences (*p* < 0.05) within group between baseline and the end.

**Table 8:** Other health effects of honey.

Honey	Dose	Duration	Subjects	Physiological Parameter	Effect	Reference
Low- and high- antioxidant buckwheat honey from the Dutch Gold company	1.5 g/kg	6 h	Healthy subjects 25.55 ± 2.30 years	Plasma phenolic concentration	↑ *	[56]
				Plasma antioxidant capacity	↑ *	
				Plasma reducing capacity	↑ *	
Manuka honey from New Zealand	Manuka honey microemulsion cream 0.5–1 cm Once a day	3 m	Patients with blepharitis 60 ± 12 years	Dry eye symptomology	↓ ^a^	[57]
				Tear film quality	↑ ^a^	
				Ocular surface quality	↑ ^a^	
				Microbial burden	↓ ^a^	
Local unpasteurized honey from Honeycomb Apiairies, Bristol and filtered pasteurized clover honey from Dutch Gold Honey Inc, Lancaster	1 tablespoonful/d	30 w	Patients with allergic rhinoconjunctivitis 45.3 years	Symptoms of rhinoconjunctivitis	=	[58]
Multiflora honey and Manuka honey UMF 20+, both produced by Comvita New Zealand Ltd.	20 g/d	Crossover 4 w each period	Healthy subjects 42–64 years	IgE level	↑	[59]
				Gut bacterial	=	
Astragalus honey made in Ashtian Region of Iran	1.2 g/kg from the 15th day to the onset of menstruation	Crossover 2 m each period	Female students with dysmenorrhea 22.01 ± 1.78 years	Pain	=	[60]
				Amount of bleeding	=	
				Satisfaction	=	

h: hour; d: day; w: week; m: month; ↑: increase; ↓: decrease; =: unchanged. * Significant differences (*p* < 0.05) within group between baseline and the end. ^a^ Significantly different (*p* < 0.05) from the control group.

**Table 9:** Main effects of honey on human health.

Condition	Subjects	Parameter	Effect	References
Cardiovascular risk factors	Healthy subjects Diabetic subjects Subjects with hyperlipidaemia	FBG	↓	[12,13,18,19,20,21]
		TG	↓	
		TC	↓	
		LDL	↓	
		HDL	↑	
Glucose tolerance	Healthy subjects Diabetic subjects	Increase in BGL	↓	[21,23,24,25]
		Increase in BIL	↓	
Alcohol metabolism	Healthy subjects	Intoxication time	↓	[27,28]
Cancer	Patients with acute myeloid leukaemia Patients with head and neck cancer	Mucositis severity	↓	[29,30,31,32,33,34,39]
		Body weight	↑	
URTIs	Children with URTIs	Cough frequency and severity	↓	[42,43,44,45,46]
		Combined symptom score	↓	
		Sleep quality	↑	
		Parent’s sleep quality	↑	
Wounds	Children with pyomyositis abscesses Women with episiotomy wound Patients with diabetes	Wound healing	↑	[51,52,53,54]
		Discharge	↓	

FBG: fasting blood glucose; TG: triglycerides; TC: total cholesterol; LDL: low-density lipoproteins; HDL: high-density lipoproteins; BGL: blood glucose level; BIL: blood insulin level; ↑: increase; ↓: decrease.

## Data Availability

Not applicable.
